# “…it is not the sickness itself that kills. It is the emotional trauma”: a qualitative study of the lived experience and systemic barriers of women with breast cancer in Nigeria

**DOI:** 10.1007/s00520-026-10377-8

**Published:** 2026-01-29

**Authors:** Eme O. Asuquo, Chigozirim Ogubuike, Omolola Salako, Adaorah Enyi, Elizabeth Abodunrin, Olusegun Biyi-Olutunde, Kate Absolom, Bassey Ebenso, Matthew J. Allsop

**Affiliations:** 1 Leeds Institute of Health Sciences, University of Leeds, Leeds, UK; 2 Department of Community Medicine, University of Port Harcourt Teaching Hospital, Port Harcourt, Rivers State Nigeria; 3 Department of Radiotherapy, College of Medicine, University of Lagos, Lagos, Nigeria; 4 Pearl Oncology Specialist Hospital, Lekki, Lagos State Nigeria; 5 Department of Radiation and Clinical Oncology, University of Port Harcourt Teaching Hospital, Port Harcourt, Rivers State Nigeria

**Keywords:** Breast cancer, Nigeria, Lived experience, Qualitative research, Supportive care

## Abstract

**Background:**

Breast cancer is the leading cancer among Nigerian women and is commonly diagnosed at an advanced stage; yet the everyday realities that influence patient outcomes and well-being remain underexplored. Understanding the lived experiences of Nigerian breast cancer patients is critical for developing culturally appropriate, supportive, and palliative care services.

**Aim:**

To explore the lived experiences, needs and coping strategies of women living with and beyond breast cancer in Nigeria.

**Design:**

Qualitative study using a constructivist–interpretivist paradigm. Semi-structured, in-depth interviews were conducted online via Microsoft Teams, audio and/or video-recorded, and transcribed verbatim. Data were analysed inductively using the Framework Method, with double coding to ensure rigour.

**Setting/participants:**

Twenty-eight women (aged 29–63 years; mean 42 years) receiving care at two oncology centres were purposively sampled between June 2024 and March 2025. Most had stage II or III disease and had lived with cancer for at least 6–12 months.

**Results:**

Four inter-linked themes described the women’s experiences: (1) “For several months, I saw myself like a ghost amongst people”—emotional, physical, economic and social upheaval following diagnosis; (2) “But I have to talk to myself, I need to encourage myself to keep going”—personal, spiritual and peer-based coping resources; (3) “I just know that there should be a lot more awareness” of the care continuum—structural, financial and informational barriers, including catastrophic out-of-pocket costs and fragmented care pathways; (4) “It is the emotional trauma”—reframing illness, personal growth and an expressed desire to support other patients. Across the themes, women stressed unmet psychosocial needs, reliance on faith communities, and the paucity of formal peer-support or counselling services.

**Conclusions:**

Participants reported navigating a complex interplay of financial toxicity, systemic delays, and profound psychosocial distress, and many described drawing on spiritual practices, self-encouragement, and peer connections to cope; several also expressed a desire to support other women by sharing advice or lived experience while going through the care pathway. Strengthening palliative and supportive care in Nigeria should prioritise: (1) financial-protection mechanisms to reduce treatment abandonment; (2) streamlined referral/navigation systems; and (3) integrated psychosocial and peer-support interventions that acknowledge spiritual coping. Further research should investigate survivor-led support models and assess the effectiveness of culturally tailored communication training for oncology teams.

**Supplementary Information:**

The online version contains supplementary material available at 10.1007/s00520-026-10377-8.

## Relevance statement

1. What is already known about the topic
  Over 70% of Nigerian women with breast cancer present at advanced stages (III–IV), with most paying out-of-pocket for treatment, leading to delays or abandonment of care.
  Fragmented referral pathways and unclear clinician communication prolong the diagnosis-to-treatment interval, often requiring repeat investigations that add financial and emotional strain.
  Psychosocial challenges such as anxiety, isolation, and fear are widespread, yet structured counselling or peer-support services are still rare in oncology care.
  While prior studies have drawn attention to survival gaps and financial burdens, there is limited qualitative evidence capturing how Nigerian women navigate these intersecting challenges throughout their cancer care experience.
2. What this paper adds
  Nearly all participants reported major treatment interruptions due to unaffordable diagnostic or chemotherapy costs.
  Unclear or absent referral information forced self-navigation, resulting in multiple biopsies and months of delay in starting treatment.
  Many women concealed their diagnosis from children or older relatives to protect them, heightening their emotional burden.
  Peer support was viewed as the most valuable yet least accessible resource, with a strong need for low-cost psychosocial interventions.
3. Implications for practice, theory, and policy
  There is a need to explore the financial protection of women living with breast cancer in Nigeria through prioritising the inclusion of cancer diagnosis, treatment, and palliative care within national insurance schemes and tightening the regulation of cancer drug supply chains.
  Breast cancer care pathways may be strengthened by developing standardised referral protocols, introducing patient navigation systems, and training healthcare providers in patient-centred communication to reduce delays and improve care continuity.
  Integrating psychosocial and community support by setting up formal peer-support networks, expanding access to culturally appropriate counselling, and partnering with faith-based and community organisations may enhance the emotional and social support available for women living with breast cancer in Nigeria.

## Background

Breast cancer is the most frequently diagnosed cancer among Nigerian women (40.5%), accounting for an estimated 32,278 new cases and 16,332 deaths in 2022 [1–3]. It is also the leading cancer in Africa and the second most common worldwide, often affecting women at a younger, pre‑menopausal age [1]. More than 70% of diagnosed breast cancer cases present at advanced stages of the disease in Nigeria [1, 2, 4, 5]. Outcomes in Nigeria mirror those across sub‑Saharan Africa. They are driven by delayed presentation, late‑stage diagnosis, and sub‑optimal treatment, compounded by a high proportion of aggressive triple‑negative tumours [2–10].

Breast cancer incidence is higher in countries with a high Human Development Index (HDI), roughly 1 in 12 women, compared with 1 in 27 in low‑HDI settings; however, mortality is disproportionately greater in low‑HDI countries, where 1 in 48 women die of the disease versus 1 in 71 in high‑HDI settings [3, 4, 7, 11]. Nigeria’s breast cancer mortality rate is one of the highest in the world, with the highest age-standardised rates (ASR) of 26.8, with a 40% survival rate compared to over 90% in high-income countries [2, 4, 7, 12, 13].

A recent systematic review showed low utilisation of multimodal therapy, with most patients receiving either single‑agent chemotherapy or a combination of surgery and chemotherapy. The study showed that mortality is highest in the first two years following diagnosis [2]. Radiotherapy, a cornerstone of breast cancer management, remains scarce in many parts of Africa. Fewer than 50% of African countries have radiotherapy facilities, and existing services represent only around one-third of optimal radiotherapy capacity. As a result, fewer than 20% of women with breast or cervical cancer receive radiotherapy [14–16]. Reported barriers include limited treatment facilities, insufficient staffing and training opportunities, and challenges with the availability, reliability, and affordability of radiotherapy units [2, 14–18].

Despite the significant burden of breast cancer in Africa, there is a notable lack of locally generated, evidence-based research to guide context-specific management strategies. As a result, many national treatment guidelines are adapted from international protocols, which may not fully align with the realities of local healthcare systems. These adapted guidelines are often implemented inconsistently across regions and facilities, leading to fragmented care and variable treatment outcomes. This inconsistency, compounded by resource limitations and workforce shortages, makes it challenging to deliver standardised and effective breast cancer care throughout the continent [4, 19, 20]. Studies on women’s experiences of surviving breast cancer in Nigeria are scarce, and existing evidence suggests that both individual-level factors and systemic healthcare challenges influence survivorship [3, 7, 13]. Some factors, such as Western lifestyle, late detection of cancer, poor access to cancer screening, poor oncology patient navigation and limited access to comprehensive cancer treatment, have been reported to cause low survival rates [3, 6, 7, 13, 18, 21–23]. Tumour laterality (increased frequency of left-side breast cancers) and patient age have also been linked to survival [24, 25]. Given the high burden of breast cancer in Nigeria, marked by late-stage presentation, limited access to multimodal treatment, and poor survival rates, there is a critical need to understand the lived experiences of women navigating these systemic and individual challenges. Existing evidence offers limited qualitative insight into how women cope with financial, emotional, and care-related barriers across the cancer continuum. To address this gap, this study aimed to explore the in-depth experiences, needs and coping strategies of women living with and beyond breast cancer in Nigeria.

## Materials and methods

### Study design

A qualitative design grounded in a constructivist–interpretivist paradigm was adopted, recognising that reality is co-constructed through individual experiences and social interactions rather than being objectively fixed [26]. In‑depth interviews were chosen for their capacity to elicit rich, personal accounts [27].

### Setting and recruitment

This study was conducted at the University of Port Harcourt Teaching Hospital, Port Harcourt, Nigeria, a public hospital and at the Pearl Oncology Specialist Hospital, Lekki, Lagos, Nigeria, a private hospital.

Port Harcourt, with a population exceeding 3.8 million, is served by the University of Port Harcourt Teaching Hospital (UPTH), a tertiary healthcare facility with a bed capacity of over five hundred. Each year, UPTH treats over 400,000 outpatients and 10,000 inpatients, and performs more than 3000 surgical procedures. The hospital’s bed occupancy rate has consistently exceeded 70% over the past 12 months. In addition to providing medical care, UPTH plays a significant role in clinical education, offering training to medical students, nurses, and other healthcare professionals [28–30]. Lagos, Nigeria’s most populous city with over 17.2 million residents, is home to Pearl Oncology Specialist Hospital in Lekki. Pearl Oncology’s vision is to be the first choice for humane cancer care and an ally in addressing cancer in Africa. Pearl Oncology prevents, treats, and cures cancer, and provides palliative care through its network of cancer centres, while advancing calmness, clarity, and hope in every patient interaction through research, education, and technology. Pearl Oncology’s practice is guided by patient-centred and leadership principles encompassing empathy, growth, partnership, and innovation [31–33].

A research nurse and assistant in each facility (who were briefed about the aims and objectives of the study) screened clinic lists and folders. They recruited women who met the inclusion criteria: aged ≥18 years, diagnosed with breast cancer for at least 6 months, and currently undergoing treatment or having completed treatment for breast cancer. Participants were recruited during their clinic visit, and only participants who agreed to take part and who were judged able to provide informed consent by the nurse were approached. The research assistant handed them a study information pack (participant information sheet and consent form). Participants were informed of their right to withdraw from the study at any time, both before and after data collection, and that any identifying information would be removed. Each participant received₦20,000 to cover transportation, refreshments, and internet access for those whose interviews were conducted from home.

### Data collection

The lead researcher (EOA) conducted all interviews via Microsoft Teams between June 2024 and March 2025. An interview guide (Appendix 2) was developed to explore psychological well-being, family involvement, and peer support, drawing on prior research conducted by the research team [3, 13] and informed by the Adversity, Restoration, Compatibility (ARC) framework (Appendix 1) [34]. The ARC framework conceptualises the psychosocial journey of individuals living with cancer as a dynamic and non-linear movement from the disruption and distress of diagnosis and treatment (Adversity), through efforts to regain emotional and relational equilibrium (Restoration), toward the re-establishment of a sense of identity, meaning, and everyday continuity (Compatibility). This framework was selected because it aligns closely with the study’s aim of understanding how women navigate emotional, social, and relational challenges across their treatment pathways. While the interview guide was not structured to map directly onto the ARC components, the framework served as a sensitising lens, shaping prompts that encouraged participants to reflect on disruption, coping, identity, and the role of supportive relationships. The experiences of participants were explored across care pathways, in this study defined as structured clinical plans that guide patient care over time and adapt to medical decisions, health outcomes, and local contexts [35, 36]. Sociodemographic and clinical data, including age, religion, income, marital status, and questions related to the stage and timing of diagnosis, were also collected from all participants prior to their interviews.

Most interviews took place online with participants in the hospital, specifically at UPTH (*n* = 16) and Pearl (*n* = 10). For participants in Pearl, some interviews were conducted at home (*n* = 2), with one participant unable to complete their interview due to illness. The research assistant at each hospital set up the participants in a quiet room and provided them with a laptop and internet connection to enable the Teams interview. They stayed available to help with any technical issues. Before the interviews began, EOA read the participant information sheet and addressed any questions and concerns the participants had. Consent forms were completed online via MS Forms, and verbal consent was obtained before the online interview began and before audio and/or video recording began. EOA also took notes. Most of the interviews were audio-recorded (*n* = 27) while one interview was video-recorded. The study was conducted in English, which is the official language of Nigeria and is generally spoken; however, the local pidgin English, which most people in Nigeria speak, was used in combination if a participant communicated in pidgin during the interview. Interviews lasted approximately 45 to 60 min for most participants, although one interview lasted 2 h. Interviews continued until data saturation was achieved and no new themes emerged.

### Data analysis

Transcripts were uploaded into NVivo and analysed using the framework analysis method [37]. Transcription was carried out by EOA and CO, who read and re-read the transcripts while listening to the audio recordings to ensure accuracy and deepen familiarity with the data. Framework analysis proceeded inductively through seven iterative stages: familiarisation, coding, developing and applying an analytical framework, indexing, charting, mapping, and interpretation [37]. Although the ARC framework informed the development of the interview guide, the analysis itself was inductive; themes were derived directly from participants’ accounts rather than being structured according to the ARC components.

To ensure coding consistency and the reliability of results, EOA began by reading and re-reading the transcripts to gain an initial understanding of participants’ perspectives. EOA read and coded ten transcripts, while a second coder (i.e. CO) coded an additional four. Coding was done iteratively, meaning codes were refined and expanded as new data were analysed. The coders compared their coding, discussed discrepancies, and reached consensus, resulting in a shared coding template that was applied to the remaining transcripts. Coding remained iterative, with new codes added as they emerged. A framework matrix in NVivo was constructed from inductive codes emerging from the data. This was then exported into Excel to organise thematic summaries. This facilitated comparison across cases, supporting identification of patterns and enabling iterative refinement of themes. Relationships between themes were explored, and higher-order categories were developed. Regular analytic meetings were held with senior team members (MJA, KA, BE) to interrogate emerging ideas, enhance reflexive interpretation, and ensure analytical rigour. Themes were finalised through discussions with MJA, ensuring they were grounded in the data and aligned with the research questions. The analytic consensus approach helped validate interpretations and reduce individual bias.

Sampling began with an initial target of 6–8 participants per site and continued until data saturation was reached. Sample size considerations were informed by Hennink et al.’s distinction between code saturation (the point at which no new codes arise) and meaning saturation (when deeper conceptual nuance is achieved) [38]. Code saturation occurred after approximately nine interviews, while a larger sample supported meaning saturation across settings.

Lincoln and Guba’s criteria of credibility, confirmability, auditability, and transferability guided the study’s trustworthiness [39, 40]. Credibility was strengthened through sustained engagement with the data, weekly debriefings between EOA and MJA and regular consultation with senior qualitative experts (KA, BE), supporting reflexivity and reducing interpretive bias [41]. Auditability was ensured through secure documentation of transcripts, coding decisions, and analytic memos, allowing for external scrutiny. Transferability was supported by providing rich contextual descriptions of study settings, participant characteristics, and thematic patterns. Although transcripts were not returned to participants for comments, direct participant extracts are used throughout the findings to illustrate and ground themes in lived experience. This iterative and collaborative approach ensured that the resulting themes were both data-driven and reflective of shared and nuanced perspectives across the sample.

## Results

### Sociodemographic and clinical characteristics of participants

Twenty-eight women were interviewed: 16 at UPTH (*n* = 16; 57.1%) and 12 at Pearl (*n* = 12; 42.9%). Most of the women were married (*n* = 26; 92.9%) and aged 29–63 years, with a mean age of 42.1 years. Most participants were diagnosed at stages II and III and had had cancer for 6–12 months. Almost all the participants had attained a tertiary education (*n* = 25; 89.3%). Most of the participants (*n* = 9; 32.1%) from UPTH had no source of income, with some (*n* = 2; 7.1%) earning less than ₦50,000 and a few (*n* = 4; 14.3%) earning between ₦50,000 and ₦150,000. The highest income earned was ₦200,000 (*n* = 1; 3.6%). While at Pearl half (6; 21.4%) earned between ₦50, 000 to ₦150, 000 with others earning between ₦300, 000 to ₦600, 000 (6; 21.4%) (Table 1).

**Table 1:** Sociodemographic and clinical characteristics of participants

Category		*n* (percentage)
Participants	*N* = *28*	
Site	*UPTH* *Pearl*	*n* = *16(57.1)* *n* = *12(42.9)*
Age	*29–63 years*	*Mean age 42.1* *(SD)* ± *3.12 )*
Marital status	*Single* *Married* *Widowed*	*n* = *1(3.6)* *n* = *26 (92.9)* *n* = *1(3.6)*
Educational level	*Secondary* *Tertiary*	*n* = *3(10.7)* *n* = *25 (89.3)*
Level of income (monthly)	*No income* *Less than* ₦*50,000* ₦*50,000–*₦1*50,000* ₦*151,000–*₦*250,000* ₦*251,000–*₦*350,000* ₦*351,000–*₦*450,000* ₦*451,000–*₦*550,000* *Above* ₦*551,000*	*n* = *9 (32.1)* *n* = *2 (7.1)* *n* = *10 (35.7)* *n* = *1(3.6)* *n* = *2 (7.1)* *n* = *1(3.6)* *2 (7.1)* *n* = *1(3.6)*
Stage of diagnosis	*0* *I* *II* *III* *IV*	*n* = *1(3.7)* *n* = *2(3.7)* *n* = *11(37.0)* *n* = *9(29.6)* *n* = *5(14.8)*
Time since diagnosis (months)	*6–12* *12–24* *24–36* *36–48*	*n* = *9(32.1)* *n* = *6(21.4)* *n* = *6(21.4)* *n* = *7(25)*
Treatment types	*Chemotherapy* *Surgery, chemotherapy* *Chemotherapy, radiotherapy* *Surgery, chemotherapy, targeted therapy* *Surgery, chemotherapy, radiotherapy* *Targeted chemotherapy* *Chemotherapy* + *Targeted Therapy*	*n* = *9* *n* = *9* *n* = *3* *n* = *1* *n* = *2* *n* = *1* *n* = *3*
Breast cancer site	*Left* *Right* *Bilateral*	*n* = *18* *n* = *7* *n* = *3*

### Themes

Our coding framework covered the range of experiences for women living with and beyond breast cancer. These experiences are discussed under four main themes: (1) “For several months, I saw myself like a ghost amongst people”—emotional, physical, economic and social upheaval following diagnosis; (2) “But I have to talk to myself, I need to encourage myself to keep going”—personal, spiritual and peer-based coping resources; (3) “I just know that there should be a lot more awareness” of the care continuum—structural, financial and informational barriers, including catastrophic out-of-pocket costs and fragmented care pathways; (4) “It is the emotional trauma”—reframing illness, personal growth and a fervent desire to support other patients. The themes are described in more detail below. See Table 2 for participants’ quotes.

**Table 2:** Participants’ quotes

Theme	Quotes
Theme 1: “For several months, I saw myself like a ghost amongst people”	***Patient 1 Pearl (Quote 1):**** Oh. I had depression; I will say it is an understatement. Is there another word that can be used? From the diagnosis, it was depressing. One thing that happened to me for several months was that I was seeing myself like a ghost amongst people. So, I have people around me, and it looks like I am just the only one there. There are people marrying and I am crying, and I am thinking that I am dead already, even though I was still breathing. It was that bad. It was that bad*
	**Patient 6 UPTH *(Quote 2):**** OK I am not doing anything; I cannot go out. I do not go out because I am still feeling pains. I cannot stand properly. I am not doing anything. Even my children. I cannot do anything for them, it is my husband that does everything in the house. I am just living under their mercy. And sometimes looking at my children, I start crying. It has not been easy. Even if I see anything I can do, I cannot do it. I do not have strength to do it even if I want to. I do not go out. I am not bringing anything to the table again the way I used to do. It looks as if there is no hope. It looks as if my dreams are dying. It looks as if I cannot continue*
	***Patient 9 UPTH (Quote 3):**** I am depressed almost all the time; you know I see myself that I am no longer who I used to be. I dissociate myself from social gatherings. I must hide myself all the time. When I hear a knock on the door, I will rush and go and wear a bra, that I never wanted to wear, you know? And this bra thing pains me. It has not been easy for me*
	***Patient 6 UPTH (Quote 4):**** I cannot go to like the open day of my children in school. Before I used to go to their school for their open day. Nobody is going there again. They have been asking of me since this year, I have not gone to their school. It has been affecting them*
	***Patient 6 UPTH (Quote 5):**** This whole thing has to do with emotions and finance, especially if you are not financially buoyant and your family is not financially buoyant It is just only God. Before the next chemo you start thinking about how you are going to get money for the next one, not just the chemo itself. You know, there are series of tests you will take or do before taking chemo. Before you know what is happening, you are spending close to ₦200,000 for just one round. It is this hard the money, there is no money. You now start thinking this also can contribute to health. It is just God, what I mean, it is just God. The little land we had, we had to sell it, for me to go for surgery, but the surgery cost ₦1.5 million because the thing affected the other breast as well, so it has not been easy and now I am even thinking about how I will continue with chemo as I am talking to you now. It has not been easy*
	***Patient 10 Pearl (Quote 6):*** *I do not eat well, even my salary, when they pay me. I cannot even eat food for up to a week. The money will finish. I have kids, I have parents. My parents, I have forgotten about them. Because what I am facing, the money is not enough for me to even feed myself well, to even eat well. So, I am not giving them anything. I cannot even afford to eat myself. They say I should not eat carbohydrates. Well, you know this Nigeria, the food we eat, rice, semovita. They say I should be eating this, eating that, and all cost good money*
	***Patient 5 UPTH (Quote 7):**** I even beg at times, for the sixth chemo, now the seventh one. I beg people. At times I pay ₦150,000, and at times ₦90,000, but I never stopped taking chemo. I have taken chemo with the help of people*
	***Patient 16 UPTH (Quote 8):**** Oh,**** i****t is only one brother that I have told, I did not tell the others. I did not tell them. I do not want people to start talking and the thing will start spreading that she has this and the way they will say it, it will look as if the person is going to die the next week, and it will make me feel bad. I do not want anything that will make me feel as if I am going to die. Because me I do not want to die, I want to survive this*
	***Patient 16 UPTH (*Quote 9):** *I have not told my office. I do not want to make it open like that. There is a way people see things here. I do not want to make it open. It is only one of my friends that I told when I was in the surgical ward that came to see me, so I did not make it open. If I take chemo maybe that week, I will call; I will take permission that week. I will take permission for the days I will stay at home because of those chemo side effects*
	***Patient 10 Pearl (Quote 10):**** I have not told so many people, my mom is 73, I have not told her, I do not think I want to tell her, because I am the one taking care of her, I pay her rent, everything, so I am not going to tell her, I do not want to go and add more to her, you know, BP (blood pressure) can start now, before you know it is beyond me*
	***Patient 1 Pearl (Quote 11):**** So, I did, I did some herbal therapies before I started chemo, but they were majorly teas and supplements, herbal supplements, and teas, yes, I took them. I also did enema. I would do coffee enema twice daily; I did that for about 3 months*
	***Patient 7 UPTH (Quote 12):**** I was first diagnosed in another hospital, but travelled home for native medication, one of my sisters had directed me to a man that can cure cancer. So, after about a month with the man, I did not see any improvement. I just zeroed my mind. I decided to come back to English medication. That is to start with this hospital. That is why I started taking English treatment, though, there after I was introduced to this networking stuff*
Theme 2: “But I have to talk to myself, I need to encourage myself to keep going”	***Patient 11 UPTH (Quote 13:**** Umm I have my husband with me, he has been a great support, my in-laws, my parents, my siblings, they have all been supportive. Giving me words of encouragement, financial support, coming to visit me. So, they have really been supportive*
	***Patient 1 Pearl (Quote 14):**** You know, so this cancer diagnosis has made me realise that you can have everything; you can trade everything on earth, but do not trade your family for anything. Family is everything. Family is key. My family has been I would say this unapologetically that there is no other family like mine. They have been so wonderful. I now realise that I did not know that I meant so much to them*
	***Patient 1 UPTH (Quote 15):*** *So, if you do not have people around you, that encourage you, you encourage yourself. But for now, I have gotten to a level like, I encourage myself. I believe that I will go through it, and I will not die. I was very depressed for some time, but I had to talk to myself, I need to encourage myself to keep going, since everybody is doing everything for me to survive, let me just survive. The way I see things, I take it like that because it really affects you emotionally*
	***Patient 4 Pearl (Quote 16):**** But I just made-up my mind from the beginning that it was a battle I had to win. It was a battle I had to channel my energy into and get my mind. Have a positive mind, positive attitude and make sure I am around people with positive thoughts. Because a lot of people, still do not understand this health challenge. I keep telling them that it is not a death sentence. So, I do not view it as such. And I always try to keep my mind occupied, so I really do not have any dark thoughts or what have you about it. I just think positive*
	**Patient 14 UPTH (Quote 17):** *OK as this thing happened initially, I was thinking I will not make it but see me here now, I am talking to you, so I talk to myself that I am strong. That God has not left me, that my God is still with me. So that is what I just tell myself every day as I wake up. You are strong, keep on moving. The Lord is with you. That is what I tell myself*
Theme 3: “I just know that there should be a lot more awareness”	***Patient 1 UPTH* (Quote 18):** *It is financial support oh if I see it, I will be happy, at least it will relieve them because they are getting tired of everything, you know, the other day I was just thinking, now if they(family) do not have money to give me for treatment again how will I cope. I am just begging God do something because the financial aspect is too much. It is too much. It is really draining people's finances and just depending on people, to feed you, pay your medical bills, pay for test’s ah it is really taxing*
	***Patient 9 UPTH (Quote 19):**** If I had a little money to buy the follow up drugs. I did not even go for the check up again because I did not have the money. You know, the check-up is now every three months. You must run this chest CT scan and so many other scans, which costs a lot. I did not have the money, so I did not go for the check up and this is the main reason it reoccurred because of those check-ups I refused to go to and the follow up drugs I was not taking. I think so. That is the reason it reoccurred. I did not follow up*
	**Patient 1 Pearl (Quote 20):** *When you ask the oncologist, “what can I eat or what should I not eat?”, they will say eat anything that appeals to you. I do not think it should be like that. You know, so we should not just eat anything. Our gut health can also be of great help. So, if we can have a good clean gut, because the gut is like our second brain. If we can have a very good gut, then it will also to be easy for us, you know, to eliminate toxins from our body and to fight different diseases as well. So whatever treatment you are getting will also give very good outcomes in the end and to the joy of everybody*
	***Patient 6 Pearl (Quote 21):**** On my own, I like reading, so I google some things. Like whenever I go for my CA 15.3 cancer serum when I get home, I read about it and I got to understand what is normal, even when the CA is high it does not mean that the breast cancer has come back. It means that you should go for check-up and consult your oncologist so those are the things. I know about it*
	***Patient 3 Pearl (Quote 22):**** Usually sometimes between the oncologist and the haematologist once they prescribe any medication, I first google it. What does it do? What are the side effects? When should I take them? I am always googling one thing or the other regarding my health challenge*
	***Patient 1 UPTH (Quote 23):**** Someone referred me to that clinic, and they were just keeping me there and we were spending money upon money until the cancer had spread, it had gone to a bad state. I then had to go to another clinic. It was the Doctor in the second clinic that referred us, that you must go to UPTH. He now referred us to this place. So, they are not doing the right thing too, if you are a doctor and somebody comes to you and it is not your area of speciality, please refer the person to the right place so that they will get the proper treatment*
	***Patient 3 Pearl (Quote 24):*** *I just know that there should be a lot more awareness even at this stage. There must be a lot more awareness and a lot of things that one can do. An explanation as to the several types of treatments that you can undergo and what you need to do post treatment is necessary. Even if it means meeting at the facility once a month or once a week and just speaking to other patients, because a lot, a lot of people I am sure they would still be terrified because really in Nigeria in the past, it was as far as we were concerned it was a death sentence. They could not even say the word cancer*
Theme 4: “It is the emotional trauma”	**Patient 1 Pearl (Quote 25):** *I could help a million and one women. I am not willing to hide my story. I have a documentary of my journey. Every day of this journey is documented. I was talking, in fact, one of the things I have found on this journey is that I have found love*
	**Patient 6 Pearl (Quote 26):** *I will be incredibly happy to share any idea I have with them to encourage them because; there is something I have seen or learnt throughout this journey. I see that it is not the sickness itself that kills. It is the emotional trauma. When you feel down, you are unhappy, you are not happy with yourself. You are afraid and fear kills, when you are afraid you will not be able to sleep, you will not be able to eat. And when you cannot sleep, you cannot eat and maybe see happiness. Definitely other sicknesses will crop in, and other things will follow. But if you have positive people around you that encourage you, that make you happy. I think I would like to give them good support if I have the opportunity*
	**UPTH patient 1 (Quote 27):** *What I think, and I always think about this is if I can get out of this. If I am free today. Ha anything I have, I will go the extra mile to support whoever is going through this, because it is not an easy journey. It is not an easy journey at all. So, if I see somebody going through it, I will look for a way: financially or emotionally that I can assist the person. I will do it because; it is a fight. It is a fight. And if you are not courageous, you will not win*
	**Patient 8 Pearl (Quote 28):** *This thing, by the grace of God, should not stop me from achieving what I want to achieve. For me, I want to read book (go to school). I am planning to go back to school. I was discussing with my dad. And secondly, I want to open a school. And by the grace of God, God help me, I will still be talking about this to women. To make them understand that through going the right way, going for treatment, they can be fine*

#### Theme 1: “For several months, I saw myself like a ghost amongst people”—emotional, physical, economic, and social upheaval following diagnosis

Women receiving breast cancer care in both hospitals (UPTH and Pearl) reported facing a complex interplay of emotional, physical, social, and economic challenges as they navigated the diagnostic and treatment continuum. Many participants described an initial period of psychological distress following their diagnosis, marked by fears related to mortality, financial burden, and concern for their families, particularly children. For some participants, this distress was compounded by spiritual uncertainty and feelings of isolation *(Quote 1, 2).*

Daily functioning was significantly affected, especially in the initial stages of treatment. Routine daily living tasks, such as bathing, cooking, and household chores, became difficult due to physical weakness or emotional fatigue. Changes in physical appearance, particularly related to hair loss and surgical outcomes, contributed to a reluctance to engage in social activities. Many women reported restricting their movements outside the home to essential errands or hospital visits *(Quote 3, 4).*

Economic hardship emerged as a critical concern. Some participants were forced to stop working or close their businesses due to the physical demands of treatment. Others changed their work routines to manage their health. However, for many, particularly single mothers or women without spousal support, continuing to work was not optional despite the strain. This shift often resulted in a loss of financial autonomy and the inability to support family and extended family members as they had in the past (Quote 5,6,7,).

Disclosure of diagnosis with families and friends was approached with caution. While a few women shared their condition with close family or religious leaders, many chose not to inform children or elderly relatives, fearing it would cause unnecessary worry. Concerns about stigma, misperceptions, and insensitive reactions were key factors influencing decisions to limit disclosure *(Quote 8,9,10).*

Complementary and alternative medicine (CAM) use was widespread among participants. All reported making dietary changes, often influenced by advice from fellow patients and/or survivors. In some cases, the women adopted strict diets consisting solely of fruits and vegetables. Some women engaged in extensive personal research on cancer, which influenced significant lifestyle and behavioural changes aimed at improving their well-being during treatment. These changes included modifications to the diet and the avoidance of synthetic products, such as perfumes and soaps, in favour of natural alternatives, such as essential oils blended with shea butter or cleansing mixtures containing apple cider vinegar, honey, and locally made black soap. This traditional black soap (volunteered by participants in Lagos), originating from the Yoruba ethnic group of Nigeria, is produced from the ash of locally harvested plants and their peels, combined with plant-based oils. These practices reflect a broader trend among patients seeking complementary approaches rooted in local knowledge and natural remedies, especially in contexts where formal/orthodox supportive care may be limited [42]. Complementary practices were commonly used to manage chemotherapy side effects; examples included body massage, localised exercises, herbal teas, dietary supplements, and mindfulness activities. Alternative therapies were sometimes used as a substitute for hospital-based care, particularly when financial barriers delayed access to formal/hospital-based treatment. These therapies were often seen as interim solutions, but many participants reported limited or no improvement, which led them to seek hospital care eventually. Specific herbal remedies mentioned included preparations known locally as “Hospital Too Far,” “Poroporo,” “Jigg small,” and “Babari,” among others *(Quote 11, 12).*

#### Theme 2: “But I have to talk to myself, I need to encourage myself to keep going”—personal, spiritual, and peer-based coping resources

The women in this study adopted a range of coping strategies to navigate the challenges of living with breast cancer, with the nature and availability of support varying widely across individuals. Study participants named seven broad sources of support.

First, family support played a vital role for many women. Emotional, spiritual, and financial support often came from partners, children, and extended family members. These family members contributed to daily household tasks, caregiving responsibilities, and, in some cases, treatment-related expenses* (Quote 13,14).*

Some of the women at Pearl Hospital had families with broader networks who could also provide links to additional clinical, psychological, and emotional support.

Second, alumnae groups of some of the women also provided financial support. However, not all women had access to such support.

Third, for those without strong family networks, neighbours often stepped in, offering both emotional comfort and practical help with domestic chores.

Fourth, religious institutions emerged as another significant source of support. Churches and faith-based communities encouraged prayer, spiritual counselling, and social visits. Some women also received material support from their pastors or members of their congregations, churches, or charities, which helped alleviate the financial burden of treatment.

Fifth, social media served as a critical tool for a few participants, particularly those who had been abandoned by their spouses. By sharing their stories online, these women were able to ask for financial help from the broader public.

In parallel, healthcare professionals in both hospitals were identified as important sources of psychological support, practical guidance during treatment, and even financial support or help with tests. For instance, some women received some funding from an NGO through Pearl to support their treatment.

Sixth, the workplace provided varying levels of support. Some women received help from empathetic colleagues who offered financial assistance, while others received ongoing salary payments even when they were unable to work or work full-time. Some women were also able to work partially from home or take some time off work during their treatment, with time off work for as long as one year in some cases. In some instances, employers reduced wages but continued to pay some partial income, helping to ease economic pressures. A few women also had their offices cover the cost of their treatment, or they obtained loans from their offices or banks to finance it. For a few women, their organisation’s health maintenance organisation (i.e. a company that provides comprehensive health insurance plans) provided a certain amount of coverage for seeing a specialist but did not cover tests or treatment. Some also accessed their husbands’ NHIS, but it offered limited service as well.

Seventh, peer networks, particularly among fellow patients and breast cancer survivors, were highlighted as an essential avenue for mutual support. These connections helped in the sharing of practical advice and firsthand experiences, contributing to a sense of solidarity and understanding.

In the absence of external support, many women drew on internal resources to cope. Religious faith was commonly described as central to coping, with personal prayer, Bible reading, and Christian music serving as tools for supporting psychological well-being. Affirmative self-talk and engagement in meaningful activities such as baking or community service visits also helped some women redirect their focus and preserve a sense of purpose beyond their illness *(Quote15,16,17).*

#### Theme 3: “I just know that there should be a lot more awareness” of the care continuum—structural, financial, and informational barriers, including catastrophic out-of-pocket costs and fragmented care pathways

Women living with breast cancer in Nigeria meet a range of structural, financial, and informational challenges that significantly affect their ability to access and sustain effective treatment. One of the most often cited issues was the economic burden associated with care. Most women reported difficulties starting or continuing treatment or following up after treatment due to the high cost of chemotherapy and related services. Many of the women from Pearl Oncology Specialist Hospital were financially more stable. They had families and networks that could support them financially, ensuring they continued their treatment once they had started. On the other hand, more of the women from UPTH were not as financially buoyant and had several interruptions in their treatment, which made their cancer worse and extended their treatment times.

Access to essential medications posed another critical barrier in UPTH. Several participants noted that chemotherapy drugs were unaffordable, with some advocating for government subsidies or the free provision of these medications. Concerns were also raised about the potential circulation of counterfeit or substandard drugs, which some women suspected might have compromised their treatment outcomes. Financial constraints extended beyond medication, affecting the ability to afford diagnostic tests needed throughout the treatment process, resulting in delayed or discontinued care *(Quote 18, 19).*

In addition to financial concerns, the need for emotional support was stressed. While some women provided emotional reassurance to their family members, they also identified a gap in structured psychosocial support for both patients and their families. The emotional demands of managing their illness in isolation, particularly in households with minimal support, underscored the importance of broader psychosocial interventions.

Peer support emerged as a particularly valued yet often inaccessible resource. Participants across both UPTH and Pearl clinics expressed a desire to connect with other women undergoing similar experiences. The lack of formal peer support networks limited opportunities for shared learning, motivation, and practical guidance during treatment.

Several participants also described challenges related to information and communication. Many were unsure where to access necessary items such as breast prostheses, and dietary advice varied widely depending on the source. The lack of standardised, reliable guidance left some women relying on anecdotal information from other patients, family members, or the internet* (Quote 20,21,22).*

Gaps in the healthcare system further complicated patients’ experiences. In some cases, women were not promptly referred to oncology departments after their initial consultations in surgical units. As a result, some only learned of oncology services through informal channels within the hospital, leading to treatment delays and the need to repeat diagnostic procedures upon referral. These system inefficiencies contributed to fragmented care and patient frustration *(Quote 23).*

Communication with healthcare providers was another area of concern. Some women reported receiving unclear or insufficient explanations about their diagnosis and treatment plan, particularly about the interpretation of test results. This created uncertainty, especially among women who lacked a system to help with decision-making. At the same time, a few participants expressed apprehension about receiving too much information, fearing that it could increase anxiety. Finally, several women highlighted delays in seeking medical care due to limited awareness about breast cancer symptoms and progression. Initial misdiagnoses or non-specialist management at earlier stages of the illness were also common, with referrals to specialist care sometimes occurring only after the disease had significantly advanced *(Quote 24).*

#### Theme 4: “It is the emotional trauma”—reframing illness, personal growth, and an expressed desire to support other patients

Several of the women interviewed expressed a strong commitment to supporting others diagnosed with breast cancer, drawing on their firsthand experiences to contribute to broader efforts in awareness and peer support. Some had already begun helping informally, while others voiced a desire to provide guidance and encouragement if they recovered fully. Their motivation was grounded in the belief that sharing lived experiences could help dispel common fears and misconceptions, particularly the notion that a breast cancer diagnosis inevitably leads to death* (Quote 25, 26, 27).*

Several participants proposed setting up organised support platforms, including suggestions for virtual safe spaces and communities such as WhatsApp groups. These spaces would serve as avenues for connection, knowledge-sharing, and emotional support, particularly for newly diagnosed patients. Many emphasised the importance of early detection and prompt medical intervention, offering advice based on their treatment timelines and outcomes. Financial help was also often cited as a critical area where support could make a substantial difference, given the significant economic challenges most participants had met during their care.

Although not all participants identified positive outcomes from their experience with breast cancer, some reflected on personal growth and shifts in perspective. For a few, the diagnosis prompted a deeper engagement with their faith and strengthened familial relationships. Recovery and return to daily life remained a primary focus for many, with aspirations ranging from resuming employment or business activities to pursuing personal goals such as marriage or expanding their families. Others aimed simply to regain their strength and resume their caregiving responsibilities* (Quote 28).*

## Discussion

This study explored the experiences of women with breast cancer in Nigeria, as there is still a low level of evidence-based research detailing the experiences and needs of women living with and beyond breast cancer in Nigeria [13, 21]. It offers one of the few qualitative accounts of Nigerian women’s journeys through the diagnostic and care pathways for breast cancer, revealing a nexus of financial hardship, systemic gaps, and substantial psychosocial distress, with participants also describing coping strategies and peer solidarity. Financial challenges emerged as a central theme in this study, reflecting the broader reality for many women with breast cancer in Nigeria. 

Consistent with previous research, most patients continue to pay out of pocket for essential components of care, including chemotherapy, radiotherapy, diagnostic tests, and medications [43]. Out-of-pocket costs reflect the broader Nigerian context, which, alongside those of Malawi, Tanzania, Uganda, and the Democratic Republic of the Congo (DRC), has some of the weakest health financing models, placing the greatest burden on households [44, 45]. As highlighted in this study, this financial burden contributes to delayed treatment initiation and premature discontinuation, particularly among those diagnosed at advanced stages. This study underscores that late-stage presentation remains common, highlighting the compounded impact of economic barriers and limited access to timely, affordable care [43, 46, 47]. Nigeria’s National Health Insurance Scheme (NHIS) offers only partial coverage for cancer diagnosis and treatment, leaving patients to shoulder the majority of costs, with most women reliant on household out-of-pocket payments [46, 48, 49]. In 2018, three-quarters of Nigeria’s health expenditure was out-of-pocket, among the highest in Africa [50]. Similar patterns across LMICs demonstrate catastrophic expenditure and treatment interruptions linked to weak insurance integration [44, 45]. Furthermore, politics has also been identified as a major reason for the chronic underfunding of cancer care in Nigeria [50].

Concerns about the medication quality and safety were prominent. Participants expressed fears of counterfeit or substandard drugs, a valid concern given that many cancer medicines in Nigeria are imported, primarily from China and India, and are often accessed through unregulated markets [51]. These findings align with those of Wilfinger et al. (2025), who reported that 17% of the medications tested across four African countries were substandard or falsified [52]. These systemic barriers underscore the need to strengthen pharmaceutical regulation, expand financial protection, and integrate cancer treatments into national insurance coverage. At the same time, partnerships with non-governmental organisations and faith-based networks could help bridge gaps in access to care.

The findings reveal significant weaknesses in referral pathways within the Nigerian healthcare system, directly affecting timely access to oncology care. Many participants described substantial delays and reliance on informal networks to learn about oncology clinics, leading to missed opportunities for early intervention. Even after referral, repeated diagnostic testing added to the financial and emotional strain. The average time from first symptom to diagnosis can extend to 9.5 months, with an additional delay of about 4 months even after initial contact with a healthcare provider [6, 10, 53, 54]. Similar patterns are observed across sub-Saharan Africa: reviews from Nigeria, Ghana, Kenya, and Uganda report average diagnostic delays of 7.4 months and nearly 5 months to treatment initiation [44, 45, 50, 51, 55]. Studies in Angola and Ethiopia indicate delays ranging from under 3 months to over 6 months [48, 53, 54, 56]. These prolonged delays significantly increase patient costs, strain health systems, and worsen survival outcomes [6, 10, 57, 58].

Communication challenges further impeded care, with many participants reporting that information from healthcare providers was unclear or insufficient. While some patients sought more detailed explanations, others preferred limited information due to fear or emotional overwhelm, underscoring the need for patient-centred, tailored communication strategies. These findings align with evidence linking poor communication, limited specialist access, and disorganised clinic systems to delays in cancer care in Nigeria [53]. In Zimbabwe, Bopoto et al. (2025) demonstrated how clinicians adapted the Baile and Buckman SPIKES model to reflect Shona cultural and linguistic norms in oncology care. Rather than applying the model verbatim, clinicians prioritised cultural sensitivity and relational harmony, positioning themselves as cultural-linguistic mediators who bridge biomedical realities with patients’ values. This adaptation underscores the complexity of navigating language, culture, and ethics in end-of-life communication [59, 60]. Across Africa, effective communication regarding diagnosis, prognosis, treatment, and end-of-life care remains inadequate, emphasising the need for provider training to enhance patient engagement and promote culturally sensitive approaches [61–63]. Strengthening care coordination will also require standardised referral protocols, patient navigation systems, and electronic referral tracking [11].

Psychosocial support was limited, leaving women to rely on religious, family, and peer networks for emotional coping. Nigeria’s context is further complicated by concerns about medication quality and uneven availability of oncology services, intensifying financial and emotional strain. Participants reported significant distress, fear, anxiety, and isolation, often managed without formal support. Despite evidence that breast cancer patients face increased risks of anxiety, depression, and social withdrawal [64–71], most women were not referred to structured support or counselling services [70]. In the absence of formal psychosocial care, spiritual coping strategies such as prayer and engagement with religious communities played a central role, consistent with findings from Angola and other studies showing that religious coping is positively associated with quality of life [56, 70–75]. Many women also shielded their families from distress, emphasising the need for culturally appropriate psychosocial interventions that integrate spiritual and community resources [76].

Support systems varied widely, with needs changing from diagnosis through survivorship [68]. While some women received substantial help from spouses, children, and extended family, others relied on neighbours, churches, or healthcare staff for emotional and practical support. Religious communities provided spiritual guidance and, in some cases, material assistance. Peer support was highly valued, yet access to structured support groups was limited, suggesting an opportunity to formalise survivor networks within cancer care. Evidence shows that social support reduces stress, mitigates treatment side effects, and improves survival [46, 73, 74]. In Ethiopia, breast cancer support groups significantly enhanced emotional resilience, treatment decision-making, and community advocacy, while reducing stigma [77]. Other studies confirm that social support lowers anxiety and depression and improves quality of life [74, 78, 79]. These findings reinforce the importance of integrating family and community networks into breast cancer management [79].

The women employed diverse coping strategies to manage the impact of their diagnosis and treatment, consistent with evidence that lower anxiety improves coping [68]. Some engaged in creative or purposeful activities such as baking or volunteering, while others focused on religious practices to sustain their hope. Dietary changes and complementary therapies were common, often guided by peer advice rather than clinical input. Alternative medicine was used when access to conventional care was delayed due to cost, though many stopped it after recognising its limited benefits.

Several participants expressed a desire to support other women facing similar challenges, often through informal mentoring or by proposing platforms, such as WhatsApp groups. Many emphasised early detection and timely medical intervention, motivated by their own experiences of delayed care or misdiagnosis. Those who had benefited from peer support were committed to “paying it forward,” thereby laying the foundation for patient-led advocacy and community education. Sharing experiences was seen as a source of emotional relief, helping women normalise their journey without burdening families; those lacking such support struggled more with family distress [76]. Peer support networks have been shown to reduce healthcare costs, provide psychosocial support, and improve the quality of life of women living with breast cancer [80].

This study provides critical insight into the lived experiences of women with breast cancer in Nigeria, revealing the complex interplay of financial, systemic, emotional, and social challenges across the care continuum. There is an urgent need to strengthen Nigeria’s health financing structure; the exclusion of cancer care from comprehensive coverage under the National Health Insurance Scheme (NHIS) leaves patients vulnerable to catastrophic expenditure. Expanding insurance to include diagnosis, treatment, and palliative care is essential. The weak and informal referral pathways highlight significant gaps in the health system. Standardised referral protocols, patient navigation services, and electronic referral systems are vital to reducing diagnostic delays and ensuring continuity of care. Provider training in patient-centred communication tailored to individual preferences and cultural contexts is essential for improving care quality and patient satisfaction. The emotional burden of breast cancer remains under-addressed; as such, integrating accessible psychosocial services, including counselling and mental health support, into cancer care is crucial. Given the centrality of spirituality, collaboration with religious and community organisations is necessary to provide culturally resonant support and enhance patient well-being.

The study highlights the vital role of peer support, informal mentoring, and survivor-led advocacy, consistent with findings from Ghana, Benin, and other Sub-Saharan African countries [81, 82]. Formalising survivor networks and support groups could strengthen psychosocial care, foster empowerment, and complement clinical services, while reducing strain on the health systems and improving survivorship outcomes. Many women incorporated lifestyle changes and complementary therapies into their care, highlighting the need for health systems to engage with local health beliefs and practices. Providing culturally competent education is essential to ensure that complementary approaches are safe and do not interfere with standard treatment.

This study, therefore, extends the ARC framework, demonstrating how socio-structural constraints, particularly cost, fragmented referral pathways, and limited psychosocial services, shape the possibilities for emotional restoration and identity compatibility in resource-constrained settings. However, the findings reflect experiences of women at a single time point and may not capture changes in coping strategies, support systems, or treatment outcomes over the cancer continuum. The study focused on participants who accessed care at these two hospitals and may differ from experiences in rural areas or at lower-tier facilities with limited oncology services. Consequently, the findings may not be generalisable to all breast cancer patients in Nigeria or across sub-Saharan Africa, given regional differences in healthcare access and cultural norms. Although educational level and monthly income were collected, employment status and rural/urban residence were not collected, limiting our ability to examine how experiences varied across these characteristics. Selection bias is possible, as participants who agreed to take part may differ from those who declined. Recall bias may also have influenced reporting, as participants could under- or overstate experiences. The use of online interviews introduced technical issues, such as poor internet connectivity, and may have constrained participation and depth of disclosure. Non-verbal cues would also have been harder to see, potentially affecting rapport and interpretation, especially since the camera was off for most of the interviews. However, the camera-off format may also have increased participants’ comfort when discussing sensitive experiences. The researcher mitigated limitations in rapport by building a connection before each interview. Finally, perspectives of healthcare providers, policymakers, and family members were not included, which may have offered complementary insights into systemic barriers and support needs.

## Conclusion

Women with breast cancer in Nigeria face interconnected financial, systemic, and emotional challenges across the care continuum. Priorities for improvement include expanding financial protection, strengthening referral and communication pathways, and integrating tailored psychosocial and peer support interventions to enhance overall well-being. This study adds to the limited body of qualitative, evidence-based research on breast cancer in Nigeria, with out-of-pocket costs highlighted as a significant barrier to prompt diagnosis and continuous treatment, compounded by factors including inadequate health insurance coverage and the risk of counterfeit medications. Weak referral systems and poor provider communication further delay care, while psychosocial and emotional needs are often neglected within formal health settings. Participants described using personal coping strategies (including faith practices and self-talk) and seeking support from family, community, and peers. Many also spoke about wanting to support newly diagnosed women, for example, by sharing information, encouragement, or practical guidance. Addressing the gaps identified through policy reforms, improved care coordination, expanded psychosocial services, and deeper community engagement is essential for improving outcomes and the quality of life for women with breast cancer in Nigeria.

## Supplementary Information

Below is the link to the electronic supplementary material.

ESM 1 Supplementary Material 1 (DOCX 16.2 KB)

ESM 2 Supplementary Material 2 (DOCX 45.1 KB)

## Data Availability

The datasets generated and/or analysed during the current study are not publicly available due to the principle of confidentiality in qualitative studies, but de-identified transcripts are available from the corresponding author upon reasonable request.

## Abbreviations

*HDI*: Human Development Index
*ARC framework*: Adversity, Restoration, Compatibility
*NHIS*: National Health Insurance Scheme
*UPTH*: University of Port Harcourt Teaching Hospital
