# Supplementary material for: “…it is not the sickness itself that kills. It is the emotional trauma”: a qualitative study of the lived experience and systemic barriers of women with breast cancer in Nigeria
Source: Support Care Cancer. 2026 Jan 29;34(2):146. doi: 10.1007/s00520-026-10377-8 (PMC12855229; doi:10.1007/s00520-026-10377-8)
Supplement: Supplementary file 1 — Supplementary Material 1 (DOCX 16.2 KB) [file 520_2026_10377_MOESM1_ESM.docx]

**Appendix 1: The ARC Framework [34]**

| **Adversity: Realizing cancer** | **Restoration: Readjusting to life with cancer** | **Compatibility** |
| --- | --- | --- |
| Life changing impact of diagnosis | Disease knowledge and experience | Benefit finding |
| Disclosing and talking about cancer | Changes in outlook | Offering peer support and willingness to help others |
| Shifts in relationship | Confidence in Healthcare | Identity for the future |
| Impact of treatment | Appraising illness and values in life | Broader perspectives of wellbeing |
| Hope for the future | Lifestyle changes |  |
| Pathway to diagnosis | Importance of social support |  |
| Effects of symptoms |  |  |
| Lack of support |  |  |
