# Supplementary material for: “…it is not the sickness itself that kills. It is the emotional trauma”: a qualitative study of the lived experience and systemic barriers of women with breast cancer in Nigeria
Source: Support Care Cancer. 2026 Jan 29;34(2):146. doi: 10.1007/s00520-026-10377-8 (PMC12855229; doi:10.1007/s00520-026-10377-8)
Supplement: Supplementary file 2 — Supplementary Material 2 (DOCX 45.1 KB) [file 520_2026_10377_MOESM2_ESM.docx]

**Breast cancer survivorship guide**

***Preamble***

Today I would like to speak to you about your experiences of living with breast cancer following treatment to ascertain existing sources of support and identify any unmet needs you have. There are no right or wrong answers. I just want to hear about your experiences. I hope the information you give today can be used to help women living with and beyond breast cancer in Nigeria. All the interviews will be anonymized afterwards. You do not have to answer all the questions. If you feel uncomfortable you can skip a question and move to the next.

If you would like to withdraw from the study at any point let me know. You can do this at any time during the interview today, or afterwards using the information on the PIS. There will be no consequences for you. The questions should last around 60 minutes. If you need to take a break at any point or want to reschedule for another day let us know. Do you have any questions before we start?

Prompt => permission to start video audio recording

| Themes | Prompt items |
| --- | --- |
| Introduce  Consent | Introduce self/ research  Explain: confidentiality, length of interview, nature of discussion (specific topics to address, free to converse, in your own words, no right or wrong answers)  Any questions  Obtain verbal consent  Start recording early |
| Research | Introduce research project and key terminologies |
| Post-treatment perceptions | *Tell me about your experiences since starting/ finishing your treatment for breast cancer*  *the question “Have you required any support relating to your condition since you completed your treatment?”*  Prompts:  *If still undergoing treatment or if finished treatment*   - Have you required any support relating to your condition since you started treatment/finished? - If yes, what were these and what support did you seek?   cash, kind – family, community, religious organizations, work   - *Did the [support mentioned] that you received meet your needs?* - *Have you sought contact with health professionals or services?* - *If yes: who were the professionals?* - Counselling - Physiotherapists - Dietitians - Menopause - *Why were they sought?* - *What was provided to you?* - *If no, can you think of any ways you may require support in the future?* - *Have you sought/used complementary and alternative services (CAM)?* - *If yes:* - *When did you seek CAM- before, during or after treatment* - *Why were they sought?* - *What was provided/what did you use?* - **Acupuncture** - **Acupressure and shiatsu** - **Aromatherapy** - **Reiki and other healing and energy therapies** - **Hypnotherapy** - **Massage** - **Reflexology** - **Meditation and mindfulness** - **Herbal therapies** - **Homeopathy** - **Yoga, Tai Chi and Chi Gung** - **Special ‘cancer’ diets and dietary supplements** - Breathing exercises for stress   *For Religion*  *Have you accessed any religious support?*   - *Available in the health facility* - *The healthcare provider asked, directed, or suggested religious support* - *Involved in religious organization*   *What* types of religious (faith/spirituality) support have you accessed and why?   - *Prayers, emotional* - *Social support- Financial, help with* *physical needs, daily tasks* - Healthcare provision   What types of religious organizations have provided support?  *If you have not sought/used complementary and alternative services (CAM), have you considered accessing any? Are there reasons for not engaging with CAM?* |
| Lifestyle after treatment | *What has changed in your daily life during/ after treatment, and do you have any long-term/after-treatment problems?*  Prompts: Do you still go to work or school?   - Flexible working hours, job reassignments/loss - Family roles - Family relationships/ *parenting issues* - Stigmatisation - Body image, *Sexual functioning,* menopause*/fertility* - Vices - Finances - Social interactions - Activities -Physical activities – swimming, exercise, diet/weight, other illnesses, other activities -driving - *Memory or concentration loss/fatigue* - Pain, - Stiffness in arms, shoulder - Skin changes that haven’t cleared - Swellings - Sleep |
| Psychological health | ***How have you felt since you started treatment/or after completing treatment?***  ***Prompt for thoughts, feelings***   - **Have you had any feelings of anxiety?** - **Worry and fear or changes in your mood** - **Excessive worrying about things going wrong** - **Fear of losing control** - **Frightening thoughts** - **Fear of illness, or death** - **Feelings of detachment or unreality** - **Racing thoughts** - **Situation avoidance** - **Poor concentration** - **Feelings of confusion** - **Becoming easily distracted** - **Poor memory** - **Hypervigilance regarding potential threats**   ***Have you had any feelings of depression?***   - **sadness and despair** - **Feeling hopeless or pessimistic** - **Feeling sad, anxious, or empty** - **irritability** - **Loss of interest in hobbies or pleasurable activities** - **Thoughts of death or suicide** - **Changes in appetite or sleep patterns**   *For any thoughts/feelings identified*   - How did you find living with these feelings? - Did you require support at any point? - What kind of support? - If yes, where did you seek the support? - Did it address your needs? - If not, how might the support have been improved for you?   ***Have you needed emergency care/help/treatment?***   - ***Are there any helplines you can call in an emergency?*** - ***If yes,*** - ***What care/help/treatment did you need?*** - ***Who did you call/what service was provided?***   *What thoughts or feelings do you have about your treatment?*   - Are they different from when you first started treatment/after treatment? - Do you think your treatment has made you better or worse?   *What thoughts or feelings do you have about breast cancer?*   - *Are they different from when you were first diagnosed/started treatment/after treatment?* - *What beliefs do you have about breast cancer?* - *Are they different from when you were first diagnosed/started treatment/after treatment?* - *Have you had/seen?* - **Psychologist** - **Psychiatrist** - **Counsellor** - **Social worker** - If yes, why? - Where did you go/how did you access the services? - Offline - Online - *If no, why not?*   Do you think you need any of these services and would you like to have them? |
| Family involvement | *Has breast cancer influenced your family/social life?*   - *Have there been concerns/issues with family or friends* - *How has your family been involved in your cancer journey*   *Have they offered you support/what kinds and have you gotten the support you need?*   - How did you ask for support - What support do you need   **Has your family needed support?**   - **What kinds of support do they need?** - **Have they gotten support** - **Who did they get support from** |
| Benefit/Future | **Has there been any positive effect of having had BC?**   - **Self-esteem** - **Better relations/ connections**   **What are/do you have any plans and projections for the future?** |
| Helping other survivors | Do you think you could help other women who have cancer?  If not, why?  If yes?  What help would you think they need?   - **Counselling** - **Physiotherapists** - **Dietitians**   What kind of help would you offer/can offer?  Do you get the help you would offer? |
| Closing | Is there anything else you would like to say that you feel is important to you?  Thank you for participating. If you need to get in touch with me afterwards or think of something else, you’d like to say my details are on the information sheet.  *If you want to speak to someone else, or you’ve found today difficult, there are also details of helplines on the PIS.*  *Would you like to be sent information about the results of this study? [note down response and preferred method of contact]. Thank you again.* |
